# The 2022 ParticipACTION Report Card on Physical Activity for Children and Youth: Focus on the COVID-19 pandemic impact and equity-deserving groups

**DOI:** 10.3389/fpubh.2023.1172168

**Published:** 2023-05-26

**Authors:** Nicholas Kuzik, Christine Cameron, Valerie Carson, Jean-Philippe Chaput, Rachel Colley, Joe Doiron, Guy Faulkner, Ian Janssen, Travis Saunders, John C. Spence, Patricia Tucker, Leigh M. Vanderloo, Mark S. Tremblay

**Affiliations:** ^1^Healthy Active Living and Obesity Research Group, Children’s Hospital of Eastern Ontario Research Institute, Ottawa, ON, Canada; ^2^Department of Pediatrics, Faculty of Medicine, University of Ottawa, Ottawa, ON, Canada; ^3^Canadian Fitness and Lifestyle Research Institute, Ottawa, ON, Canada; ^4^Faculty of Kinesiology, Sport, and Recreation, University of Alberta, Edmonton, AB, Canada; ^5^Health Analysis Division, Statistics Canada, Ottawa, ON, Canada; ^6^Independent Practitioner, Dartmouth, NS, Canada; ^7^School of Kinesiology, University of British Columbia, Vancouver, BC, Canada; ^8^Department of Public Health Sciences, School of Kinesiology and Health Studies, Queen’s University, Kingston, ON, Canada; ^9^Department of Applied Human Sciences, University of Prince Edward Island, Charlottetown, PE, Canada; ^10^School of Occupational Therapy, Western University, London, ON, Canada; ^11^ParticipACTION, Toronto, ON, Canada

**Keywords:** advocacy, policy, health communication, child health, knowledge translation

## Abstract

**Introduction:**

The ParticipACTION Report Card on Physical Activity for Children and Youth is the most comprehensive national assessment of physical activity and related behaviors, characteristics, and opportunities for children and youth. The 2022 Report Card assigned grades based on data gathered during the COVID-19 pandemic to reflect this extraordinary time-period in Canada. Further, while not graded, efforts were made to summarize key findings for early years children and those identifying as: having a disability, Indigenous, 2SLGBTQ+, newcomers to Canada, racialized, or girls. The purpose of this paper is to summarize the 2022 ParticipACTION Report Card on Physical Activity for Children and Youth.

**Methods:**

The best available physical activity data captured during the whole COVID-19 pandemic was synthesized across 14 different indicators in four categories. The 2022 Report Card Research Committee assigned letter grades (i.e., A–F) based on expert consensus of the evidence.

**Synthesis:**

Grades were assigned for: Daily Behaviors (*Overall Physical Activity*: D; *Active Play*: D−; *Active Transportation*: C−; *Organized Sport*: C+; *Physical Education*: Incomplete [INC]; *Sedentary Behaviors*: F; *Sleep*: B; *24-Hour Movement Behaviors*: F), Individual Characteristics (*Physical Literacy*: INC; *Physical Fitness*: INC), Spaces and Places (*Household*: C, *School*: B−, *Community and Environment*: B), and Strategies and Investments (*Government*: B−). Compared to the 2020 Report Card, the COVID-19 specific grades increased for *Active Play* and *Active Transportation*; and decreased for *Overall Physical Activity*, *Sedentary Behaviors*, *Organized Sport*, and *Community and Environment*. There were many data gaps for equity-deserving groups.

**Conclusion:**

During the COVID-19 pandemic, the grade for *Overall Physical Activity* decreased from a D+ (2020) to a D, coinciding with decreases in grades reflecting fewer opportunities for sport and community/facility-based activities as well as higher levels of sedentary behaviors. Fortunately, improvements in *Active Transportation* and *Active Play* during COVID-19 prevented a worse shift in children’s health behaviors. Efforts are needed to improve physical activity for children and youth during and post-pandemic, with a greater emphasis on equity-deserving groups.

## 1. Introduction

The benefits of physical activity for school-aged children and youth (5–17 years) are well known and span many health and well-being indicators (e.g., adiposity, cardiometabolic biomarkers, physical fitness, bone health, quality of life/well-being, motor skill development, psychological distress, pro-social behavior) (1). However, in Canada a D+ grade was assigned for Overall Physical Activity in the 2020 ParticipACTION Report Card on Physical Activity for Children and Youth (2)—based on 61% not meeting the physical activity recommendation of ≥60 min/day of moderate- to vigorous-intensity physical activity (MVPA) (3, 4). Reflecting the depth and breadth of benefits combined with the low prevalence of sufficient physical activity for children and youth, public health efforts have been employed to improve physical activity and related behaviors, characteristics, and opportunities (e.g., MVPA, sleep, physical fitness, school environment) (5–7).

ParticipACTION^1^ i.e., a & b is a Canadian non-profit organization aiming to help people in Canada move more (8). One avenue for pursuing this mission is knowledge mobilization and active dissemination efforts, such as the ParticipACTION Report Card on Physical Activity for Children and Youth. The Report Card aims to synthesize and disseminate research “*to drive social action for policy and behavior change relating to physical activity among children and youth*” (9). While the prevalence of insufficient physical activity is high for children and youth in Canada, *Overall Physical Activity* Report Card letter grades have either improved slightly or remained the same from 2007 to 2020 (2, 10).

On 11 March 2020, the World Health Organization (WHO) declared COVID-19 a global pandemic leading to a drastic shift in the ways children and youth could access physical activity opportunities. Public health officials determined that it was necessary to implement measures to curb transmission of the virus including lockdowns, closures, and reduced capacity limits for schools, sports programs, recreation facilities, and outdoor activity spaces (e.g., playgrounds, parks) (11). While the pandemic and implemented public health measures impacted all Canadians, some groups may have experienced a disproportionate impact based on pre-existing inequalities (12). Thus, the 2022 ParticipACTION Report Card on Physical Activity for Children and Youth represents a unique glimpse into a period defined by COVID-19. The purpose of this paper is to summarize the development of the 2022 Report Card and the subsequent findings in the context of COVID-19 and with a focus on equity-deserving groups (those who face barriers to equal access, resources, and opportunities within society due to systemic discrimination and disadvantage) (13, 14).

## 2. Methods

The 2022 Report Card was produced by ParticipACTION, with the Healthy Active Living and Obesity Research Group (HALO) at the Children’s Hospital of Eastern Ontario (CHEO) Research Institute^2^ playing a critical role in the research and development. Representatives from ParticipACTION and HALO had the first guiding meeting in January 2021. At this meeting, 10 Canadian experts for the Report Card Research Committee (RCRC) were proposed and subsequently invited (100% success rate) to steer the Report Card along with the Report Card Chief Scientific Officer, Research Manager and Lead Author, and Project Manager, for a total of 13 members. The invited experts were recruited for their content expertise in different domains of children’s physical activity, geographic dispersion, and sector representation (i.e., academic, government, and non-government). The first RCRC meeting was held in June 2021 to discuss the structure and content of the Report Card (e.g., included indicators, benchmarks). Subsequently, data were gathered and synthesized from multiple sources, including national datasets and the best available peer-reviewed research for the RCRC to appraise at a meeting held in December 2021. The challenges of synthesizing COVID-19-specific data were discussed, and an additional meeting was arranged to appraise the evidence relevant to the pandemic. In March 2022, the final RCRC meeting was held. The 2022 Report Card development process was similar to previous years (2, 15, 16) with the exceptions of some pandemic influenced changes (e.g., shorter and virtual meetings).

The evidence appraisal conducted at the RCRC meetings consisted of reviewing and discussing the gathered data, assigning letter grades to the synthesized data across 14 indicators, within 4 categories—Daily Behaviors (*Overall Physical Activity, Active Play, Active Transportation, Organized Sport, Physical Education, Sedentary Behaviors, Sleep, 24-Hour Movement Behaviors*), Individual Characteristics (*Physical Literacy, Physical Fitness*), Spaces and Places (*Household, School, Community and Environment*), and Strategies and Investments (*Government*). Each indicator consisted of 1–7 benchmarks that were established *a priori*, and grades were assigned based on the average percent score (e.g., percent of children meeting the *Overall Physical Activity benchmark*), or consensus appraisal, across all benchmarks (see Table 1). If the RCRC determined insufficient evidence was available an incomplete grade was assigned (INC).

**Table 1:** Conversion of percent scores to report card letter grades.

											
A+	94%–100%	B+	74%–79%	C+	54%–59%	D+	34%–39%			INC	Insufficient data to assign a grade
A	87%–93%	B	67%–73%	C	47%–53%	D	27%–33%	F	0%–19%		
A−	80%–86%	B−	60%–66%	C−	40%–46%	D−	20%–26%				

National surveys and device-measured data are the preferred sources to inform grades. Specific to this Report Card, grades were assigned based on data collected during the COVID-19 pandemic, ranging from April 2020 to Spring 2022. COVID-19 disrupted data collection for many nationally representative surveys, resulting in some smaller and less representative data sources in the 2022 Report Card compared to previous years. Surveys used within this Report Card can be found in Table 2.

**Table 2:** Key data sources.

Data source	Sample description	Indicators
Canadian Community Health Survey (CCHS), Statistics Canada, Custom analysis (17, 18)^a^	*n* = ~5,000, 12–17 years	Overall physical activity
Cohort Study for Obesity, Marijuana Use, Physical Activity, Alcohol Use, Smoking and Sedentary Behavior, Custom analysis uwaterloo.ca/compass-system	*n* = 133 schools, Grades 9–12	Overall physical activity
		Organized Sport
		Sedentary behaviors
		Sleep
		24-h movement behaviors
ParticipACTION COVID-19 Surveys (PCS), ParticipACTION (19, 20)	*n* = ~1,500, 5–17 years	Overall physical activity
		Sedentary behaviors
		Sleep
		24-h movement behaviors
National Physical Activity Measurement (NPAM) study (21)	*n* = 86, 4–17 years with a disability	Overall physical activity
		Sedentary behaviors
		Sleep
		24-h movement behaviors
Parent Survey on Physical Activity and Sport sub-sample, Canadian Fitness and Lifestyle Research Institute (CFLRI), Custom analysis cflri.ca/settings-based-studies	*n* = ~6,000, 5–17-years	Active play
		Active transportation
		Organized
		Sport
		Household
		Community and environment
Active Transportation and Independent Mobility Study, Custom analysis pathresearch.wordpress.com/projects/	*n* = ~2,300, 7–12 years	Active transportation
		Sedentary behaviors
		Sleep
		24-h movement behaviors
Opportunities for Physical Activity at School Study sub-sample, CFLRI, Custom analysis cflri.ca/settings-based-studies	*n* = ~500, school administrators	School
Survey of Physical Activity Opportunities in Canadian Communities, CFLRI, Custom analysis cflri.ca/settings-based-studies	*n* = ~900, communities with at least 1,000 residents	Community and environment

^a^CCHS results were synthesized from Watt and Colley (17) for the Overall Physical Activity grade and Colley and Watt (18) for Overall Physical Activity in equity-deserving groups.

Another unique feature of the 2022 Report Card was a focus on equity-deserving groups. While grades were assigned to the general population of children and youth in Canada aged 5–17 years, deliberate efforts were made to also summarize key findings for those identifying as: having a disability, Indigenous, 2SLGBTQ+ (Two-Spirit, Lesbian, Gay, Bisexual, Transgender, Queer or Questioning and additional sexual orientations and gender identities), newcomers to Canada, racialized, or girls. While early years children (<5 years) were not considered an equity-deserving group, they were considered a population of interest, and results were summarized together. Equity-deserving groups, or populations of interest, were selected based on RCRC consensus for key groups of youth to discuss in relation to physical activity and COVID-19. However, it was recognized that the selected groups were not all-encompassing and other unrepresented groups deserving attention should be explored in the future.

## 3. Synthesis

### 3.1. Daily behaviors

#### 3.1.1. Overall physical activity: D

**Figure 1:**
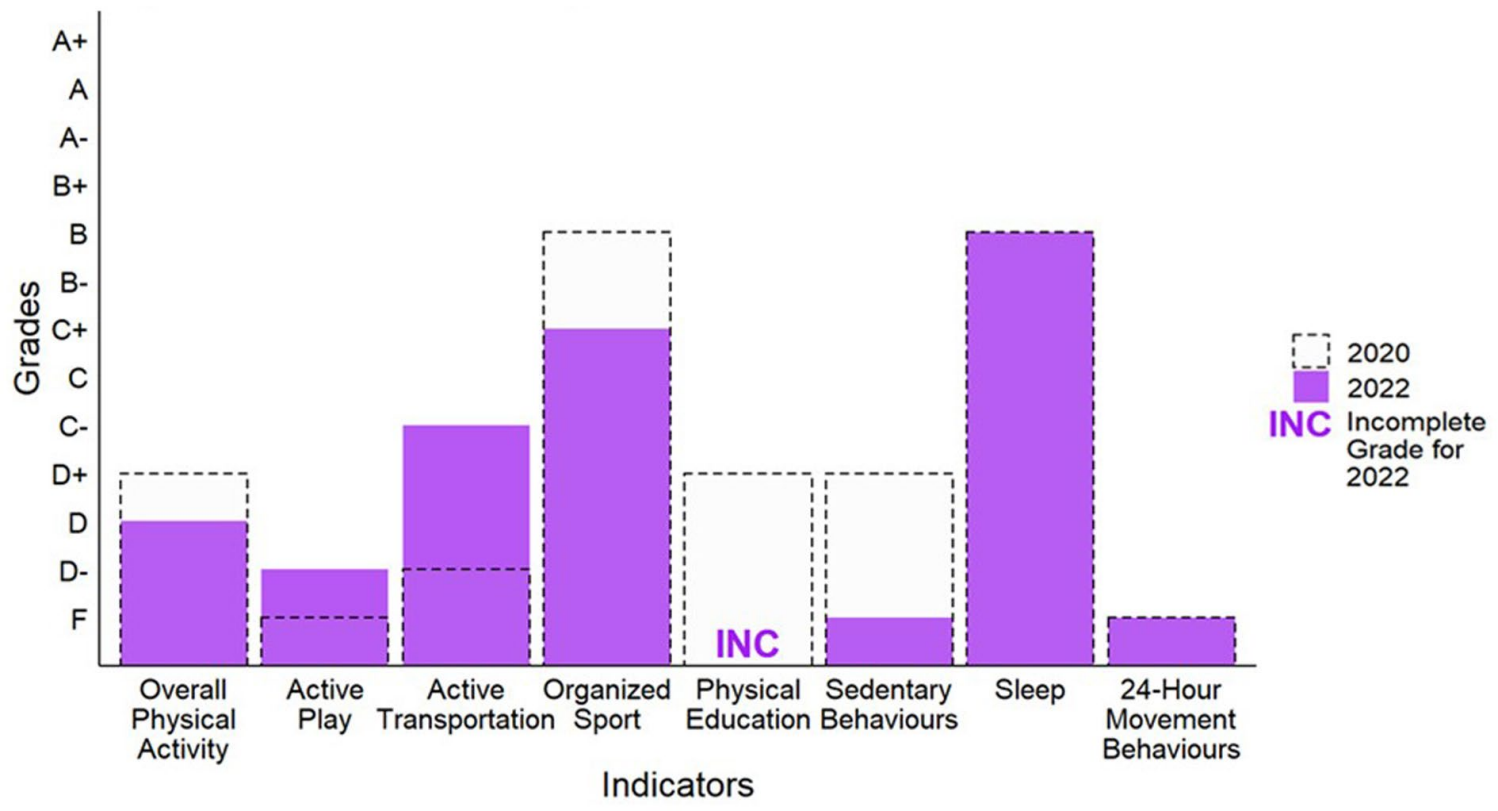
Grades for daily behaviors in 2020 and 2022.

According to the Canadian Community Health Survey (CCHS), Cohort Study for Obesity, Marijuana Use, Physical Activity, Alcohol Use, Smoking and Sedentary Behavior (COMPASS), and ParticipACTION COVID-19 Surveys (PCS), the average percent of children and youth meeting the physical activity recommendations (i.e., MVPA recommendation: at least 60 min/day, on average, of MVPA; muscle and bone strengthening recommendation: muscle and bone strengthening activities at least 3 days/week) within the Canadian 24-Hour Movement Guidelines for Children and Youth (4) according to self- or parent-report was 28%. Within the CCHS, it was estimated that the percent of youth (12–17 years) meeting the MVPA recommendation decreased from 51% pre-pandemic (fall 2018) to 37% during the COVID-19 pandemic in fall 2020. In the COMPASS study, 58% of youth in grades 9–12 met the MVPA recommendation, while 34% met the MVPA and muscle and bone strengthening recommendations. In the PCS, 24% of children 5–11-years-old and 13% of youth 12–17-years-old met the MVPA recommendation at the start of the pandemic (April 2020), compared to 18% of children and 12% of youth in October 2020. The 28% average resulted in a D grade–a decrease from the D+ grade assigned in 2020 (Figure 1) (2).

##### 3.1.1.1. Equity-deserving groups

**Figure 2:**
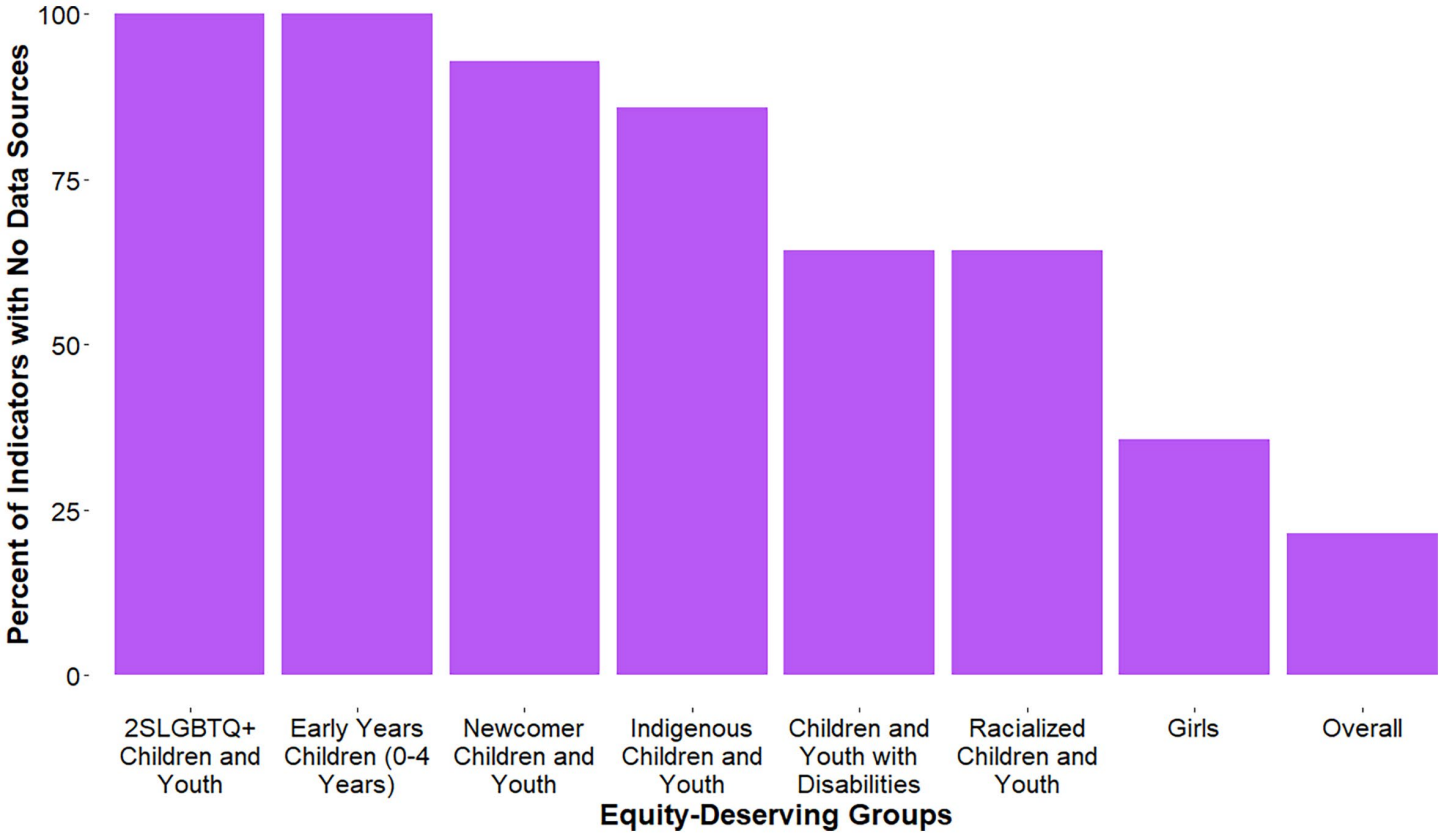
Percent of indicators with no results, for each equity-deserving group.

For the *Overall Physical Activity* indicator, COVID-19-specific results were available for equity-deserving groups within the National Physical Activity Measurement (NPAM) study (children and youth with disabilities), CCHS (Indigenous youth, newcomer youth, racialized youth, and girls), COMPASS (racialized children and youth, and girls), and PCS (girls). COVID-19-specific results were not found for other equity-deserving groups (Figure 2).

In May 2020, 7% of children and youth with disabilities met the MVPA recommendation, compared to 6% in November 2020 in the NPAM study. Within the CCHS, the percent of youth (12–17 years) meeting the MVPA recommendation pre-pandemic (fall 2018) and during the pandemic (fall 2020) was 67% and 38% for Indigenous youth, 56% and 35% for newcomer youth, 47% and 34% for racialized youth, and 46% and 35% for girls (55% and 40% for boys), respectively.

#### 3.1.2. Active play: D−

According to the Parent Survey on Physical Activity and Sport (PSPAS), 25% of children and youth (5–17 years) had an average of >2 h/day of total time engaged in indoor and outdoor unstructured play. This included physical activity and sport at home, outdoor unstructured play in the community, outdoor time in spaces such as parks and green spaces, and outdoor time at school. Based on 25% meeting this benchmark, a D− grade was assigned. This is an increase from the F grade assigned in 2020 (Figure 1).

##### 3.1.2.1. Equity-deserving groups

For *Active Play*, COVID-19-specific results were available for girls within the PSPAS (Figure 2). Specifically, 24% of girls and 25% of boys had an average of >2 h/day of total time engaged in indoor and outdoor unstructured play.

#### 3.1.3. Active transportation: C−

From the Active Transportation and Independent Mobility Study (ATIM) and PSPAS, the average percent of children and youth typically using active transportation to get to and from places (e.g., school, park, mall, friend’s house) was 41%. Results of the PSPAS survey indicated 46% of children and youth (5–17 years) either solely used active transportation, or partially used active transportation (active transportation in combination with non-active modes to or from school, such as walking or bicycling at least part of the way to school). Within the ATIM study, the percent of 7–12-year-old children that used active transportation to get to school was 37% in December 2020 and 40% in June 2021. Based on 41% of children and youth meeting this benchmark, a grade of C− was assigned. This is an increase from the D− grade assigned in 2020 (Figure 1).

##### 3.1.3.1. Equity-deserving groups

For *Active Transportation*, COVID-19-specific results were reported for girls in the PSPAS and the ATIM studies (Figure 2). Within the PSPAS, 43% of girls and 48% of boys either solely or partially used active transportation to or from school. The percentage of girls in the ATIM study using active transportation to get to school was 38% in December 2020 (36% of boys) and 42% in June 2021 (38% of boys).

#### 3.1.4. Organized sport: C+

Based on data from COMPASS and PSPAS, the average percent of children and youth participating in organized sport was 57%. Within COMPASS, 50% of youth in grades 9–12 participated in an organized sport program during the pandemic. The PSPAS survey indicated 63% of children and youth (5–17 years) participated in sport, compared to 74% pre-COVID-19. Based on 57% meeting this benchmark, a grade of C+ was assigned, which is a decrease from B in 2020 (Figure 1).

##### 3.1.4.1. Equity-deserving groups

For *Organized Sport*, COVID-19-specific results were available within COMPASS (racialized youth, and girls), and the PSPAS (girls; Figure 2). The percent of racialized youth in COMPASS who participated in organized sports during the pandemic was 37% for Asian students, 52% for Black students, 40% for Latin American/Hispanic students, 46% for other/multiple and 52% for White students. Forty-seven percent of girls in COMPASS participated in organized sport during the pandemic, compared to 54% of boys. Sixty percent of girls and 66% of boys participated in organized sport during the COVID-19 pandemic according to the PSPAS.

#### 3.1.5. Physical education: INC

No COVID-19-specific data were available to assign a grade for the *Physical Education* indicator, thus it is deemed Incomplete (INC; Figures 1, 2).

#### 3.1.6. Sedentary behaviors: F

In the COMPASS, ATIM, and PCS, 18% of children and youth met the sedentary behavior recommendation within the Canadian 24-Hour Movement Guidelines for Children and Youth of no more than 2 h of recreational screen time per day (4). For the COMPASS study, 3% of youth in grades 9–12 met the screen time recommendation from November 2020 to June 2021. Within the PCS, 16.5% of children (5–11 years) and 6.6% of youth (12–17 years) met the screen time recommendation at the start of the pandemic (April 2020), compared to 35.4% of children and 16.5% of youth in October 2020. In the ATIM study, 23% of 7–12-year-old children met the screen time recommendation in December 2020 and 25% in June 2021. Based on an average of 18%, an F grade was assigned for the *Sedentary Behavior* indicator, which is a decrease from the 2020 Report Card grade of D+ (Figure 1).

##### 3.1.6.1. Equity-deserving groups

For the *Sedentary Behaviors* indicator, COVID-19-specific results were available for equity-deserving groups within the NPAM study (children and youth with disabilities), COMPASS (racialized children and youth, and girls), ATIM (girls), and PCS (girls; Figure 2). In May 2020, 7% of children and youth with disabilities in the NPAM study met the screen time recommendation, compared to 17% in November 2020. The percent of racialized youth in COMPASS meeting the screen time recommendation during the pandemic was 4% for Asian students, 4% for Black students, 2% for Latin American/Hispanic students, 4% for other/multiple, and 3% for White students. Four percent of girls in COMPASS met the screen time recommendation during the pandemic, compared to 3% of boys. Further, at the start of the pandemic, 16% of girls 5–11-years-old (17% of boys) and 8% of girls 12–17-years-old (5% of boys) met the screen time recommendation, compared to 38% of girls 5–11-years-old (33% of boys) and 17% of girls 12–17-years-old (16% of boys) later on in the pandemic (October 2020). Finally, within the ATIM study, the percentage of girls meeting the screen time recommendation was 24% in December 2020 (21% of boys) and 26% in June 2021 (23% of boys).

#### 3.1.7. Sleep: B

In the COMPASS, ATIM, and PCS the average percent of children and youth meeting the sleep duration recommendation (5–13-year-olds, 9–11 h/night on average; 14–17-year-olds: 8–10 h/night on average) within the Canadian 24-Hour Movement Guidelines for Children and Youth (4) was 60%. For the COMPASS study, 58% of youth in grades 9–12 met the sleep duration recommendation from November 2020 to June 2021. Within the PCS, 69.9% of children (5–11 years) and 72.1% of youth (12–17 years) met the sleep duration recommendation at the start of the pandemic (April 2020), compared to 54.9% of children and 59.5% of youth in October 2020. In the ATIM study, the percent of 7–12-year-old children meeting the sleep duration recommendation was 55% in December 2020 and 53% in June 2021. While the average of 60% equates to a B− grade, members of the RCRC reached consensus on a B grade based on less gradable data sources available compared to previous years, which may have skewed the percentages lower. Further, there is research indicating that sleep has increased or remained the same throughout the pandemic (22). This aligns with the B grade in 2020 (Figure 1).

##### 3.1.7.1. Equity-deserving groups

For the *Sleep* indicator, COVID-19-specific results were available for equity-deserving groups within the NPAM study (children and youth with disabilities), COMPASS (racialized children and youth, and girls), ATIM (girls), and PCS (girls; Figure 2). In May 2020, 59% of children and youth with disabilities in the NPAM study met the sleep duration recommendations, compared to 62% in November 2020. The percent of racialized youth in COMPASS meeting the sleep duration recommendation during the pandemic was 49% for Asian students, 46% for Black students, 51% for Latin American/Hispanic students, 52% for other/multiple, and 61% for White students. Sixty percent of girls in COMPASS met the sleep duration recommendation during the pandemic, compared to 58% of boys. Further, at the start of the pandemic 73% of girls 5–11-years-old (68% of boys) and 74% of girls 12–17-years-old (71% of boys) met the sleep duration recommendations, compared to 59% of girls 5–11-years-old (52% of boys) and 62% of girls 12–17-years-old (57% of boys) later on in the pandemic (October 2020). Finally, within the ATIM study, the percentage of girls meeting the sleep duration recommendation was 57% in December 2020 (53% of boys) and 56% in June 2021 (51% of boys).

##### 3.1.7.2. 24-hour movement behaviors: F

In the COMPASS, ATIM, and PCS an average of 5% of children and youth met the combined physical activity, screen time, and sleep duration recommendations within the Canadian 24-Hour Movement Guidelines for Children and Youth (4). For the COMPASS study, from November 2020 to June 2021, 1% of youth in grades 9–12 met all recommendations. Within the PCS, 5% of children (5–11 years) and 1% of youth (12–17 years) met all the recommendation at the start of the pandemic (April 2020), compared to 5% of children and 2% of youth later on in the pandemic (October 2020). The percent of 7–12-year-old children meeting the sleep recommendation was 10% in December 2020 and 13% in June 2021 in the ATIM study. Based on the 5% average of children and youth meeting this benchmark, an F grade was assigned, consistent with the F in 2020 (Figure 1).

##### 3.1.7.3. Equity-deserving groups

For the *24-Hour Movement Behaviors* indicator, COVID-19-specific results were available for equity-deserving groups within the NPAM (children and youth with disabilities), COMPASS (racialized children and youth, and girls), ATIM (girls), and PCS (girls; Figure 2). In both May 2020 and November 2020 for the NPAM study, 0% of children and youth with disabilities met all the recommendations within the 24-Hour Movement Guidelines. For all girls, boys, and racialized groups of youth in COMPASS, 1% met all 24-Hour Movement Guideline recommendations (including the muscle- and bone-strengthening recommendation) during the pandemic. For the PCS, at the start of the pandemic, 3% of girls 5–11-years-old (7% of boys) and 1% of girls 12–17-years-old (1% of boys) met all 24-Hour Movement Guideline recommendations, compared to 5% of girls 5–11-years-old (4% of boys) and 1% of girls 12–17-years-old (2% of boys) in October 2020. Within ATIM, the percentage of girls meeting all 24-Hour Movement Guideline recommendations was 11% in December 2020 (9% of boys) and 12% in June 2021 (13% of boys).

### 3.2. Individual characteristics

#### 3.2.1. Physical literacy: INC

**Figure 3:**
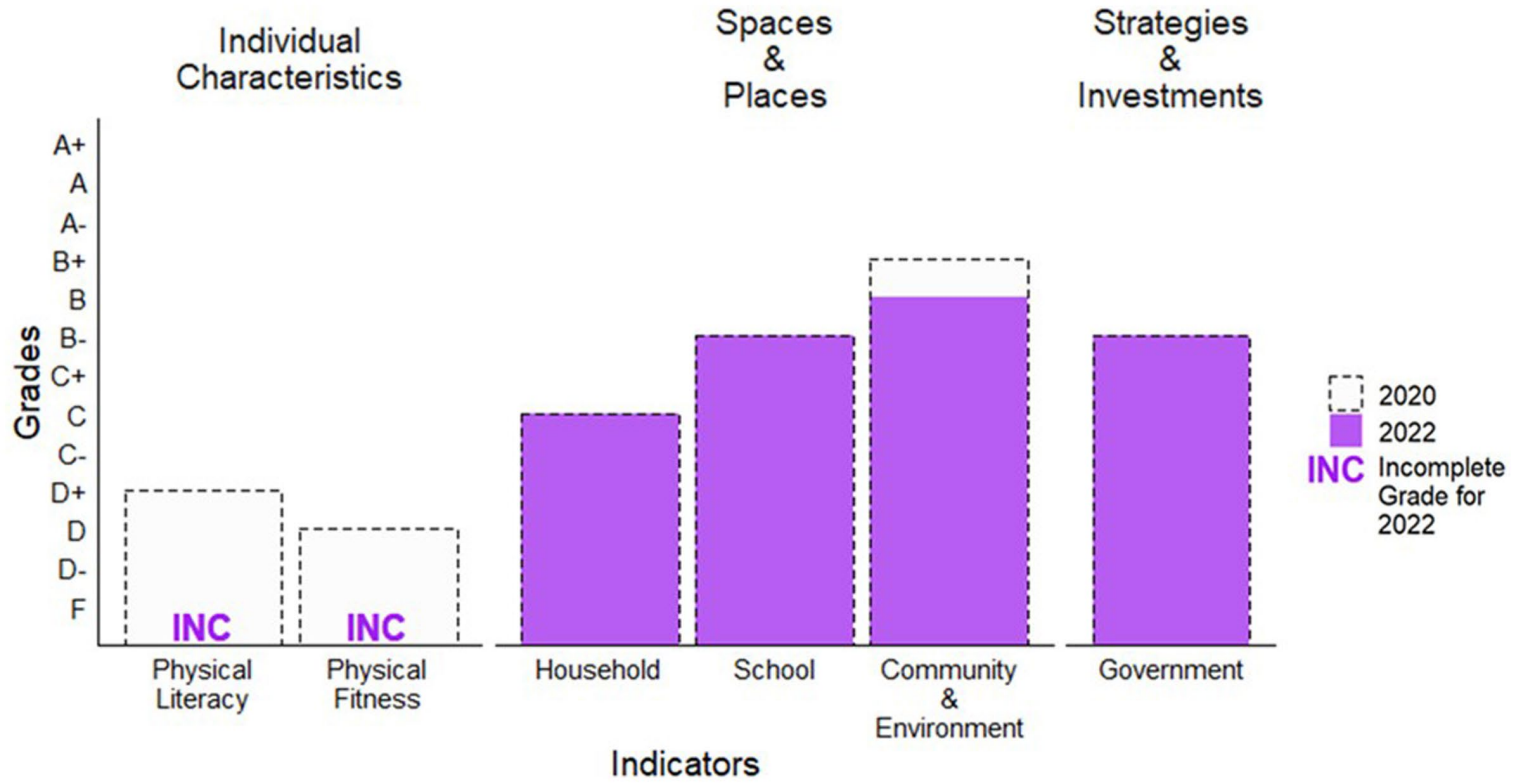
Grade comparisons beyond daily behaviors in 2020 and 2022.

No COVID-19-specific results were available to assign a grade; thus, the grade for the *Physical Literacy* indicator is Incomplete (INC; Figures 2, 3).

#### 3.2.2. Physical fitness: INC

No COVID-19-specific results were available to assign a grade; thus, the grade for the *Physical Fitness* indicator is Incomplete (INC; Figures 2, 3).

### 3.3. Spaces and places

#### 3.3.1. Household: C

According to the PSPAS, an average of 53% was calculated from the *Household* indicator benchmarks. Specifically, 53% of parents responded they facilitated physical activity and sport opportunities for their children often or very often, with examples of facilitation including: transporting, spectating, encouraging outdoor play regularly, encouraging their children to participate in play instead of screens, placing limits on screen time, and playing active games or sports with their children. Based on the 53% meeting this benchmark, a C grade was assigned—the same grade assigned in 2020 (Figure 3).

##### 3.3.1.1. Equity-deserving groups

For the *Household* indicator, COVID-19-specific results were available for girls within the PSPAS (Figure 2). Within the PSPAS, no significant gender differences were observed for the *Household* indicator benchmarks, though according to parent report 74% of boys and 69% of girls were encouraged to play outdoors regularly.

#### 3.3.2. School: B−

The average percent across the *School* indicator domains of policies, human resources, facilities, partnerships, and other programming was 64% based on the Opportunities for Physical Activity at School Study (OPASS). For policies, 66% of schools had active school policies. For human resources, 67% of schools had a physical education specialist or teacher with at least one elective credit in physical education, and >65% of students were taught physical education by a physical education specialist. For facilities, 61% of schools indicated that their outdoor and indoor facilities for physical education and physical activity met students’ needs well or very well. For partnerships, 60% of schools indicated that they had agreements with municipalities or sport organizations to share facilities or resources and programming. Finally, for other programming, 68% of schools indicated that intramural activities, inter-school activities, and other physical activity outings were available to their students. Based on the 64% average score, a B− grade was assigned, which is consistent with the 2020 Report Card (Figure 3).

##### 3.3.2.1. Equity-deserving groups

For the *School* indicator, COVID-19-specific results were not available for any equity-deserving groups (Figure 2).

#### 3.3.3. Community and environment: B

The average across the *Community and Environment* indicator domains of policies; human resources; facilities and infrastructure; partnerships; and programming for children, youth, and families was 70% according to the Survey of Physical Activity Opportunities in Canadian Communities (SPAOCC) and one benchmark from the PSPAS. For policies, 27% of communities had a formal plan for parks, recreation, physical activity and sport, or active transportation. For human resources, 65% of communities indicated they had sufficient human resources supporting physical activity. For facilities and infrastructure, 81% of communities indicated having at least one amenity promoting active transportation (public transport, crossing guards, school safety zones, etc.); 74% of communities reported having designated bike lanes on roads or multi-purpose trails; 75% of communities reported having at least one family-friendly amenity (i.e., family changing facilities, washrooms at parks, drinking fountains, childcare services); 79% of parents in the PSPAS reported that some or many facilities in their community (public, commercial, playgrounds, parks, other community facilities) were available to participate in physical activity or sport. For partnerships, 66% of communities indicated they had agreements in place with schools, school boards, or sport organizations to share facilities or resources and programming. For programming for children, youth, and families, 92% of communities reported having programming targeted to children, youth, and families. Based on the 70% average across the benchmarks, a B grade was assigned for the *Community and Environment* indicator. The B grade for 2022 is a decrease from the B+ grades assigned in 2020 (Figure 3).

##### 3.3.3.1. Equity-deserving groups

For the *Community and Environment* indicator, COVID-19-specific results were not available for any equity-deserving groups (Figure 2).

### 3.4. Strategies and investments

#### 3.4.1. Government: B−

The RCRC graded the *Government* indicator using the benchmarks: (1) evidence of leadership and commitment in providing physical activity opportunities for all children and youth, (2) allocated funds and resources for the implementation of physical activity promotion strategies and initiatives for all children and youth, and (3) demonstrated progress through the key stages of public policy making (i.e., policy agenda, policy formation, policy implementation, policy evaluation and decisions about the future). Specific data synthesized included federal, provincial, and territorial budgets, as well as initiatives related to children’s physical activity [see full summary (13)]. The RCRC reached consensus on a B− grade for the 2022 *Government* indicator, which is consistent with the 2020 Report Card grade (Figure 3).

##### 3.4.1.1. Equity-deserving groups

For the *Government* indicator, synthesized information (13) included COVID-19-specific results for children and youth with disabilities, Indigenous children and youth, and girls (Figure 2).

## 4. Discussion

During the COVID-19 pandemic, the 2022 ParticipACTION Report Card on Physical Activity for Children and Youth grade for *Overall Physical Activity* was a D, a decrease from a D+ in the 2020 Report Card. This is the first decrease in the *Overall Physical Activity* grade since 2007, where it had either remained unchanged or improved up to 2020 (2, 10). Canadian national results from the CCHS demonstrated that physical activity decreased for children and youth, which was related to decreased access to schools and opportunities for sports and recreation (17). Unsurprisingly, the *Overall Physical Activity* grade drop coincides with grade drops for *Organized Sport*, *Community and Environment*, and *Sedentary Behaviors*.

It is important to highlight that for some children and youth, positive changes to the landscape of physical activity opportunities were apparent. Specifically, increases in the grades for *Active Transportation* and *Active Play* were observed when comparing the 2022 to the 2020 grades. Aligning with the WHO’s recommendation of “*whenever feasible, consider riding a bicycle or walking*” (23) for maintaining physical distancing and promoting physical activity during transport, some cities in Canada (e.g., Montreal, Halifax) expanded or allocated car-free spaces for active transportation (24, 25). The beginning of the pandemic included restrictions on outdoor spaces such as playgrounds, coinciding with parental perception of lower levels of children’s outdoor play in April 2020 compared to pre-pandemic levels (11). As the public health guidelines evolved, restrictions to outdoor spaces were relaxed and in October 2020 levels of outdoor play improved for Canadian children and youth compared to April 2020, although outdoor play levels were still considered lower than pre-pandemic levels (20). While parents felt children’s level of outdoor play was lower than pre-pandemic levels in April and October 2020, according to the PSPAS survey, 25% of children and youth (5–17 years) had an average of >2 h/day of total time engaged in indoor and outdoor unstructured play—thus, warranting the D− minus grade for *Active Play*, an improvement from the F in 2020. These improvements are important considering the positive association between outdoor time and adherence to the 24-Hour Movement Guidelines for children and youth during COVID-19 (26).

The distribution of improvements in physical activity opportunities during COVID-19 (i.e., *Active Transportation* and *Active Play*) could be considered one-sided, as the car-free spaces were generally in areas with fewer visible minority populations and fewer households with children (24). Further, increases in outdoor time were more likely for children in higher income families, living in a house (not apartment), and living in lower population-density neighborhoods (27). As well, the unfavorable trends in behaviors seen generally (e.g., *Overall Physical Activity*, *Organized Sport*, *Sedentary Behaviors*) may have been exacerbated for equity-deserving groups given the pre-existing barriers to physical activity. For instance, for some equity-deserving groups (e.g., children and youth with disabilities, newcomers to Canada) community resources, structured programming, and school-based activities (21, 28) are key facilitators of physical activity, and COVID-19 pandemic restrictions left limited accessible alternatives (21). Thus, COVID-19 recovery plans should address inequalities for health, and health behaviors, as recommended by the Chief Public Health Officer of Canada (12).

Reflecting on the challenges that COVID-19 presented for children and youths’ physical activity opportunities, Canada should strive toward a recovery plan that draws on the momentum seen for *Active Transportation* and *Active Play*. Public health officials should advocate for increased access to the car-free and outdoor spaces that likely prevented the *Overall Physical Activity* grade from decreasing even further during COVID-19. However, all health promotion efforts need to be considered from an equity lens, or the risk of widening inequalities for physical activity and related behaviors, characteristics, and opportunities will remain an ongoing issue. Further, support is needed for surveys with large sample sizes, across robust sets of physical activity related variables, that allow for meaningful examinations of sub-groups during the recovery period of COVID-19.

### 4.1. Strengths and limitations

A strength of this review was the RCRC, a group of international leading experts in children’s physical activity from across Canada representing the academic, government, and non-government sectors. While there was a lack of data on equity-deserving groups, the effort to synthesize available evidence could be considered a strength. We hope this Report Card serves as a catalyst for future equity-based children and youth physical activity research and initiatives, such as the 2022 Canadian Para Report Card on Physical Activity for Children and Adolescents with Disabilities (29). However, limitations of this work must be acknowledged. While the selected benchmarks and indicators undergo expert appraisal and consensus by the RCRC, it is essential to continue exploring alternative and additional approaches to ensure the most essential concepts related to children’s physical activity are synthesized. For instance, while recreational screen time is a common benchmark for sedentary behavior (4, 30), a more fulsome understanding of sedentary behavior may be achieved by examining additional benchmarks (e.g., sitting time, types of recreational screen time). The synthesized data represents multiple time-points during COVID-19. The restrictions to physical activity opportunities during COVID-19 were not temporally or geographically homogenous. Temporally, restrictions were generally stricter during the first wave of the pandemic and began gradually relaxing in later waves. Geographically, differences existed in restrictions, infection rates, and movement behaviors across provinces and territories (31). COVID-19 also imposed restrictions on data collection that decreased the quantity and quality of grade-informing evidence compared to previous years. For instance, this Report Card relied on less nationally representative data and no device-measured data were available. Finally, by broadly focusing on multiple equity-deserving groups we may have missed important nuances within groups (e.g., disability impairment types and severity) and between groups (e.g., intersections between gender and race). However, the possibility of examining important nuances within groups and intersections among groups requires sufficiently powered and representative population-level data, and standardized definitions for group categorization (e.g., International Classification of Functioning, Disability and Health) (32, 33).

## 5. Conclusion

The impact of COVID-19 on children and youth included decreased physical activity and increased sedentary behaviors—both important health-related behaviors. Improvements in *Active Transportation* and *Active Play* during COVID-19 may have prevented an even greater negative shift in children’s health-related behaviors and overall well-being. Future efforts should continue enhancing equitable access to car-free and outdoor spaces. Additionally, surveys with large sample sizes capable of conducting meaningful comparisons for all children and youth, including equity-deserving groups, are needed to better understand the COVID-19 impacts and recovery efforts.

## Author contributions

NK synthesized the results and drafted the manuscript. All authors contributed to the report card conception, design, grading; and contributed to the article and approved the submitted version.

## Funding

This study was supported by the Open access publication fees were supported through a Canadian Institutes of Health Research (CIHR) Planning and Dissemination Grant.

## Conflict of interest

JD was employed by ParticipACTION.

The remaining authors declare that the research was conducted in the absence of any commercial or financial relationships that could be construed as a potential conflict of interest.

## Publisher’s note

All claims expressed in this article are solely those of the authors and do not necessarily represent those of their affiliated organizations, or those of the publisher, the editors and the reviewers. Any product that may be evaluated in this article, or claim that may be made by its manufacturer, is not guaranteed or endorsed by the publisher.

## Footnotes

^1^ www.participACTION.com

^2^ https://www.haloresearch.ca/
